# Giant hiatal hernia

**DOI:** 10.11604/pamj.2020.37.86.26141

**Published:** 2020-09-24

**Authors:** Danilo Coco, Silvana Leanza

**Affiliations:** 1 Department of General Surgery, Ospedali Riuniti Marche Nord, Pesaro, Italy,; 2 Department of General Surgery, Carlo Urban Hospital, Jesi, Ancona, Italy

**Keywords:** Giant hiatal hernia, diaphragmatic hernia, volvulus

## Image in medicine

A Giant hiatal hernia (GHH) is a type III hernia with a sliding and para esophageal component such as > 30% of the stomach, colon, spleen or pancreas due a chronic positive pressure on the diaphragmatic hiatus. Surgical repair requires hernia sac excision, tension-free repair and Nissen fundoplication. Recurrence rates range between 2% and 12%. A 77 years old Caucasian woman presented to the ED with significant thoracic pain, vomiting and bradycardia (<50 bpm). She presented a medical history of atrial fibrillation and anticoagulant therapy. She reported a traumatic rupture of the sternum 5 years ago. Her vital signs were: blood pressure 130/70 mmHg, respiratory rate 40 breaths/minute, heart rate 129 beats/minute and temperature superior of 36 C. Oxygen saturation was 85% on room air. The abdominal examination was normal. Thoracic examination reported reduced vesicular murmur. Laboratory evaluation revealed high leukocytosis with a white blood cell (WBC) count of 16 per mm^3^. Arterial blood gases (ABG) demonstrated metabolic acidosis. Computed tomography revealed a giant hiatal hernia with stomach, ileum and colon in thoracic cavity. The patient was immediately started intravenous (IV) fluids of 2l in 6 hours, Foley and jugular catheter vein cannulation to support main arterial pressure and urine output. The patient was discussed for surgical operations.

**Figure 1:**
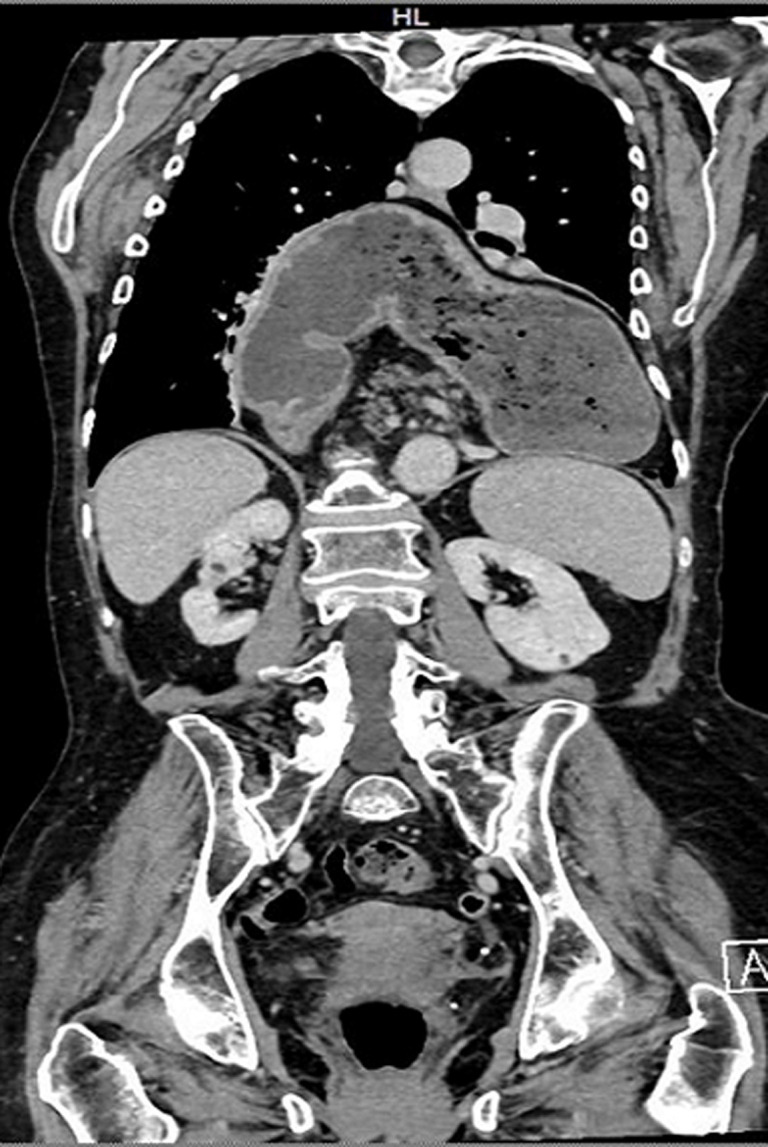
computed tomography revealed a giant hiatal hernia with stomach, ileum and colon in thoracic cavity

